# Accuracy of a Single, Heparin-Calibrated Anti-Xa Assay for the Measurement of Rivaroxaban, Apixaban, and Edoxaban Drug Concentrations: A Prospective Cross-Sectional Study

**DOI:** 10.3389/fcvm.2022.817826

**Published:** 2022-03-17

**Authors:** Tamana Meihandoest, Jan-Dirk Studt, Adriana Mendez, Lorenzo Alberio, Pierre Fontana, Walter A. Wuillemin, Adrian Schmidt, Lukas Graf, Bernhard Gerber, Ursula Amstutz, Cedric Bovet, Thomas C. Sauter, Lars M. Asmis, Michael Nagler

**Affiliations:** ^1^Department of Epidemiology, Maastricht University, Maastricht, Netherlands; ^2^Department of Clinical Chemistry, Inselspital, Bern University Hospital and University of Bern, Bern, Switzerland; ^3^Department of Medical Oncology and Hematology, University Hospital Zurich, Zurich, Switzerland; ^4^University of Zurich, Zurich, Switzerland; ^5^Institute for Laboratory Medicine, Kantonsspital Aarau AG, Aarau, Switzerland; ^6^Service and Central Laboratory of Hematology, CHUV, Lausanne University Hospital, Lausanne, Switzerland; ^7^Division of Angiology and Hemostasis, Geneva University Hospitals, Geneva, Switzerland; ^8^Division of Hematology and Central Hematology Laboratory, Cantonal Hospital of Lucerne and University of Bern, Bern, Switzerland; ^9^Institute of Laboratory Medicine and Clinic of Medical Oncology and Hematology, City Hospital Zurich, Zurich, Switzerland; ^10^Center for Laboratory Medicine, St. Gallen, Switzerland; ^11^Clinic of Hematology, Oncology Institute of Southern Switzerland, Bellinzona, Switzerland; ^12^Department of Emergency Medicine, Inselspital, Bern University Hospital, Bern, Switzerland; ^13^Centre for Perioperative Thrombosis and Haemostasis, Zurich, Switzerland

**Keywords:** diagnostic accuracy, anti-Xa assay, laboratory monitoring, direct oral anticoagulants, rivaroxaban

## Abstract

**Background:**

Applying a single anti-Xa assay, calibrated to unfractionated heparin to measure rivaroxaban, apixaban, and edoxaban would simplify laboratory procedures and save healthcare costs.

**Aim:**

We hypothesized that a heparin-calibrated anti-Xa assay would accurately measure rivaroxaban, apixaban, and edoxaban drug concentrations and correctly predict clinically relevant drug levels.

**Methods:**

This analysis is part of the Simple-Xa study, a prospective multicenter cross-sectional study conducted in clinical practice. Patients treated with rivaroxaban, apixaban, or edoxaban were included. Anti-Xa activity was measured using the Siemens INNOVANCE^®^ Heparin assay. Drug concentrations were determined using ultra-high performance liquid chromatography-tandem mass spectrometry (LC-MS/MS). Cut-off levels were determined in a derivation dataset (50% of patients) and sensitivities and specificities were calculated in a verification dataset (50% of patients).

**Results:**

Overall, 845 patients were available for analysis. Correlation coefficients (r_*s*_) between the heparin-calibrated anti-Xa assay and drug concentrations were 0.97 (95% CI 0.97, 0.98) for rivaroxaban, 0.96 (0.96, 0.97) for apixaban, and 0.96 (0.94, 0.99) for edoxaban. The area under the receiver operating characteristics curve (ROC) was 0.99 for all clinically relevant drug concentrations. In the verification dataset, the sensitivity was 94.2% (95% CI 90.8–96.6) for 30 μg L^–1^, 95.8% (92.4–98.0) for 50 μg L^–1^, and 98.7% (95.5–99.9) for 100 μg L^–1^. Specificities were 86.3% (79.2–91.7), 89.8% (84.5–93.7), and 88.7% (84.2–92.2), respectively.

**Conclusion:**

In a large prospective study in clinical practice, a strong correlation of heparin-calibrated anti-Xa measurements with LC-MS/MS results was observed and clinically relevant drug concentrations were predicted correctly.

## Graphical Abstract

Visual summary.

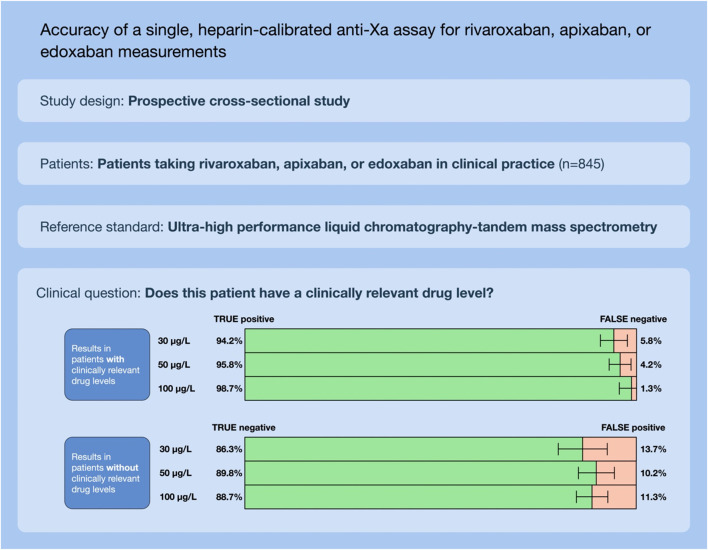

## Highlights

**What is known about this topic?**

- Applying a single anti-Xa assay to measure rivaroxaban, apixaban, and edoxaban would simplify laboratory procedures and save healthcare costs.
- It remains unclear if this can be achieved using a single anti-Xa assay, calibrated to unfractionated heparin.

**What does this paper add?**

- We conducted a prospective multicenter cross-sectional study including 845 patients taking rivaroxaban, apixaban, or edoxaban in clinical practice.
- The association between heparin-calibrated anti-Xa measurements and LC-MS/MS results was strong for all drugs.
- Clinically relevant drug levels were predicted correctly.

## Introduction

The proportion of patients taking direct oral anticoagulants (DOAC) for the prevention and treatment of thromboembolic diseases is rapidly increasing (1, 2). These patients occasionally face clinical situations with a high bleeding risk such as accidents, urgent surgery, and thrombolysis because of acute stroke (3–5). Besides, relevant DOAC drug concentrations contribute to massive bleeding of any cause (3, 6, 7). In addition, unresponsive or demented patients present to the emergency department, and knowledge of anticoagulant treatment is essential in the management. Rapid determination of DOAC drug levels in these situations supports clinical decisions regarding reversal agents, and deferral of interventions (8–10). Additionally, accumulation in the case of renal and/or hepatic failure or even overdosing can be detected (11, 12). Thus, determination of DOAC drug levels in special clinical situations is recommended by major scientific societies such as the International Society on Thrombosis and Haemostasis (9). Furthermore, it might potentially save health care costs associated with the clinical situations mentioned above (13).

Ideally, a simple laboratory test that accurately determines DOAC plasma levels would be available and implemented in various healthcare settings in a 24/7 service (3). Routine coagulation tests are neither sensitive nor specific in the detection of DOAC (10, 14–16). Various anti-Xa assays are available that measure rivaroxaban, apixaban, or edoxaban concentrations using drug-specific calibration curves (17). However, these tests are still not widely implemented because it is laborious and expensive to provide three different tests (3). We and other authors hypothesized that a single heparin-calibrated anti-Xa assay would be sufficient to accurately and efficiently determine rivaroxaban, apixaban, and edoxaban drug levels (18–20). An essential advantage of this method is that heparin-calibrated assays are already available even in smaller laboratories. Thus, implementing a single-calibration anti-Xa assay for unfractionated heparin, low molecular weight heparin, rivaroxaban, apixaban, and edoxaban would improve laboratory procedures but also the care of patients treated with DOAC. Recently, we demonstrated that a universal, LMWH-calibrated assay can accurately measure DOAC drug levels (21). However, it remains unclear if this can be achieved using a universal heparin-calibrator intended to detect unfractionated heparin and low molecular weight heparins.

With the present multicenter cross-sectional study, we aimed to assess whether the Siemens INNOVANCE^®^ Heparin anti-Xa assay would accurately measure rivaroxaban, apixaban, and edoxaban drug concentrations and correctly predict clinically relevant drug levels. The primary focus was on the clinically significant concentration range between 0 and 300 μg L^–1^.

## Materials and Methods

### Study Design, Setting, and Population

**FIGURE 1:**
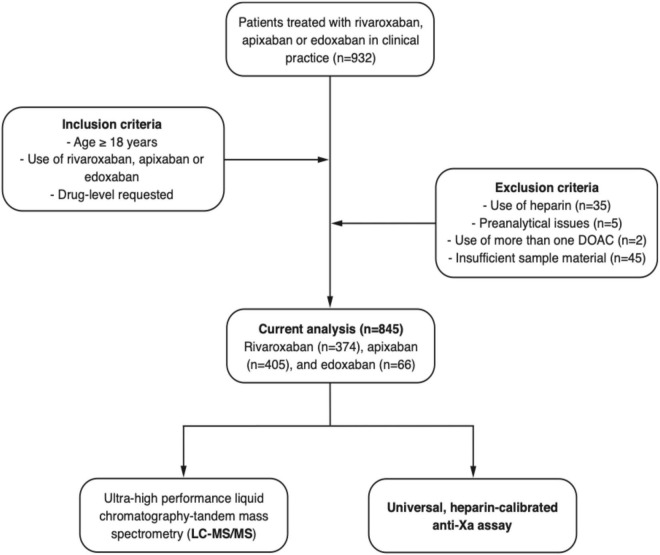
Flow of the patients. A prospective, multicentre cross-sectional study was conducted to test the accuracy of a heparin-calibrated anti-Xa assay for the measurement of rivaroxaban, apixaban, or edoxaban plasma levels in clinical practice.

We conducted a prospective multicenter cross-sectional study including patients in nine hemostasis laboratories affiliated to Swiss tertiary hospitals. Patients treated with rivaroxaban, apixaban, or edoxaban in clinical practice were included between 2018 and 2019 (Figure 1, CONSORT flow diagram). Inclusion criteria were (a) 18 years or older, (b) use of rivaroxaban, apixaban, or edoxaban (c) DOAC drug-level requested, and (d) signed general informed consent, if required by local authorities. Exclusion criteria were (a) refused general informed consent, (b) use of heparin, (c) preanalytical issues, (d) use of more than one DOAC, and (e) insufficient sample material. Samples were collected regardless of the time of last drug intake, covering the full range of drug levels observed in clinical practice. Ultra-high performance liquid chromatography-tandem mass spectrometry (LC-MS/MS) measuring rivaroxaban, apixaban, and edoxaban was used as a reference standard (16, 21). The study was approved by the local ethics committees and all hospitals gave local feasibility approval. If required, patients signed a general consent to use their samples and data before enrolment at the respective study center. The study was conducted in accordance with the declaration of Helsinki.

### Data Collection and Handling of Samples

Patient characteristics including age, sex, and the DOAC used (rivaroxaban, apixaban, or edoxaban) were collected in a secured REDCap database. Protocols were implemented at all institutions detailing blood-drawing procedures to ensure adequate pre-analytical conditions (22). Venous blood samples were drawn in plastic syringes containing 1 mL trisodium citrate (0.106 mol L^–1^) per mL of blood (i.e., S-Monovette^®^ Citrate, Sarstedt, Nümbrecht, Germany). Samples were centrifuged according to an established scheme (1,500 *g* for 10–15 min, or 3,137 *g* for 7 min), and aliquots were frozen immediately and stored at −80°C until transportation (22). Samples were shipped on dry ice in one batch per site to the central laboratory and delivered within 3 to 4 h. Samples were kept frozen until the determination of laboratory tests without any freeze-thaw cycle. Laboratory test results were exported automatically to avoid typing errors.

### Determination of the Anti-Xa Activity

The Siemens INNOVANCE^®^ Heparin anti-Xa assay was selected for determining the anti-Xa activity. This one-stage chromogenic assay is designed to measure the drug level of unfractionated heparin (UFH) as well as low molecular weight heparin (LMWH) (INNOVANCE^®^ Heparin anti-Xa assay application sheet; Siemens Healthineers, Marburg, Germany). The reagent contains dextran sulfate but not exogenous antithrombin. The instructions provided by the manufacturer were strictly followed. In brief, a five-step calibration curve was applied using the calibrators provided by the manufacturer (0.0, 0.42, 0.86, 1.26, and 1.67 U/ml). Samples were rapidly thawed and gently mixed at 37°C. Patient plasma was pre-diluted (1:2) and added to the reagent containing coagulation factor Xa and a chromogenic substrate. The formation of paranitroaniline was quantified optically at a wavelength of 405 nm. The measurements were conducted in batches on an Atellica COAG 360 analyzer (Siemens Healthineers, Marburg, Germany) (23). Measurements were performed blinded to the LC-MS/MS test results.

### Determination of the Drug Concentration by Liquid Chromatography-Tandem Mass Spectrometry

Liquid chromatography-tandem mass spectrometry was used for the quantification of rivaroxaban, apixaban, edoxaban, and M4 metabolite of edoxaban. For protein precipitation and analyte extraction, plasma acetonitrile:water 1:1 (v/v), extraction buffer (MassTox TDM Series A, Chromsystems, Gräfelfing, Germany), and precipitation reagent (MassTox TDM Series, Chromsystems, Gräfelfing, Germany) containing the isotope labeled internal standards (13C6 rivaroxaban, 13CD3 apixaban, 13CD2 edoxaban; provided by the manufacturers) were added to the plasma. Afterward, the samples were vortexed and centrifuged at 14,000 rcf and 20°C for 4 min. The supernatant was diluted with water:methanol 8:2 (v/v) and stored at 10°C until analysis. The calibrators and QCs were prepared in pooled plasma (Innovative Research, Novi, MI, United States). The extracted samples were analyzed using reversed-phase chromatography (Cortecs UPLC C18 column, 2.1 × 75 mm, 1.7 μm, Waters) on a triple quadrupole mass spectrometer (Xevo TQ-S, Waters, Milford, CT, United States) coupled to a UPLC Acquity I-Class system (Waters, Milford, CT, United States). Edoxaban M4 concentration was summed up with the edoxaban for further analysis.

### Statistical Analysis

Variables were described using proportions and percentages or median and interquartile range (IQR) as appropriate. The normality of the data was assessed visually and using a Q-Q plot. The accuracy of the anti-Xa assay was determined by calculating the Spearman’s correlation coefficient in relation to the plasma concentration as measured by LC-MS/MS. A correlation coefficient of rs ≥ 0.95 was considered as accurate (alternative hypothesis), and a correlation of rs ≤ 0.6 was regarded as inadequate (null hypothesis). The Deming regression was used to describe the linear relationship and a modified Bland-Altman plot (ratios were used due to different scales) was created to observe a potential bias over the spectrum of measurements (9). Systematic differences were analyzed by calculating the mean difference and the SD to compute 95% limits of agreement for every level of measurements (average difference ± 1.96 standard deviation of the difference) (24). To assess the diagnostic accuracy of the anti-Xa assay, we determined the sensitivity and specificity of detecting 30, 50, and 100 μg/L, representing clinically relevant drug levels. The dataset was randomly split in half, and the cut-offs of the new test were obtained by a ROC curve analysis in the derivation dataset. As an internal validation, we repeated the ROC curve analysis in the verification dataset and calculated sensitivities and specificities regarding clinically relevant drug levels. An area under the receiver-operating characteristics (ROC) curve ≥ 0.95 and sensitivities/specificities of at least 90% were regarded as adequate. A power analysis for a one-sample correlation test was conducted with a power of 0.9 and an alpha of 0.05. Since many patients were taking rivaroxaban and apixaban, and only a few edoxaban, 932 patients were included until data saturation was reached. Sensitivity analyses considering samples below 3.34 U ml^–1^ only were conducted. All statistical analyses were performed using RStudio (1.3. 1093-1); figures were created using Prism 8 (GraphPad Software, Inc., La Jolla, CA, United States).

## Results

### Patient Characteristics

Overall, 932 patients were included in this prospective multicenter cross-sectional study; a detailed flow chart is given in Figure 1. From this study population, 35 patients were excluded because of heparin use, five patients due to preanalytical issues, two patients using more than one DOAC, and 45 patients due to insufficient sample material. Eventually, samples of 845 patients were used for the current analysis. Of these, 374 patients used rivaroxaban, 405 apixaban, and 66 edoxaban. The median age was 76 years old (IQR, 66 to 82 years), and 42.9% of the patients were female, see Table 1.

**TABLE 1:** Characteristics of patients treated with rivaroxaban, apixaban or edoxaban (*n* = 845).

	Patients treated with				
	Rivaroxaban	Apixaban	Edoxaban	All	Missing data
**Patients** *(n/%)*	374 (44.3)	405 (47.9)	66 (7.8)	845 (100)	0
**Age** *(years; median/IQR)*	74	78	75	76	96
	(63-83)	(63-83)	(58-82)	(66-82)	
**Sex** *(n/%)*					8
Male	209 (56.0)	233 (58.4)	36 (55.4)	478 (57.1)	–
Female	164 (44.0)	166 (41.6)	29 (44.6)	359 (42.9)	–

*N, number. IQR, interquartile range.*

### Association Between Anti-Xa Activity and Drug Concentration

**FIGURE 2:**
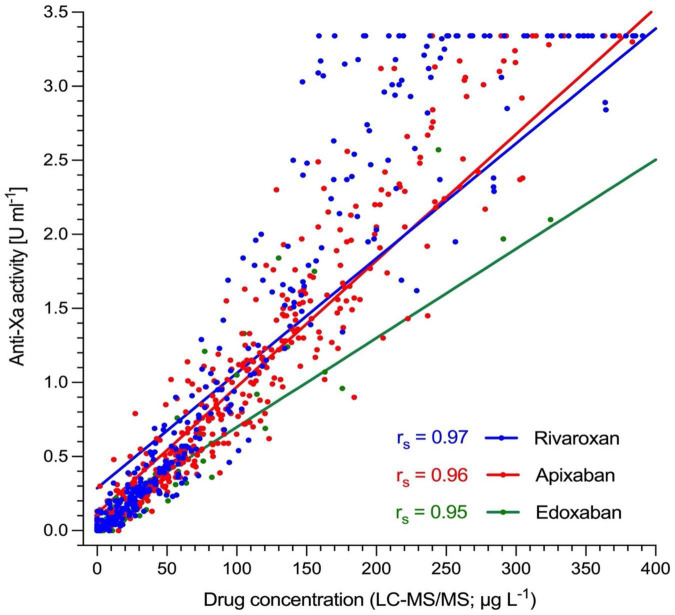
Association of heparin-calibrated anti-Xa measurements with drug concentration in 845 patients taking rivaroxaban, apixaban, or edoxaban in clinical practice. Ultra-performance liquid chromatography-tandem mass spectrometry (LC-MS/MS) was used to determine drug levels. Spearman’s correlation coefficient (r_*s*_) was 0.97 for rivaroxaban (95% CI 0.97–0.98), 0.96 for apixaban (0.95–0.97), and 0.96 for edoxaban (0.94–0.99). The overall r_*s*_ was 0.97 (95% CI, 0.97–0.98).

**FIGURE 3:**
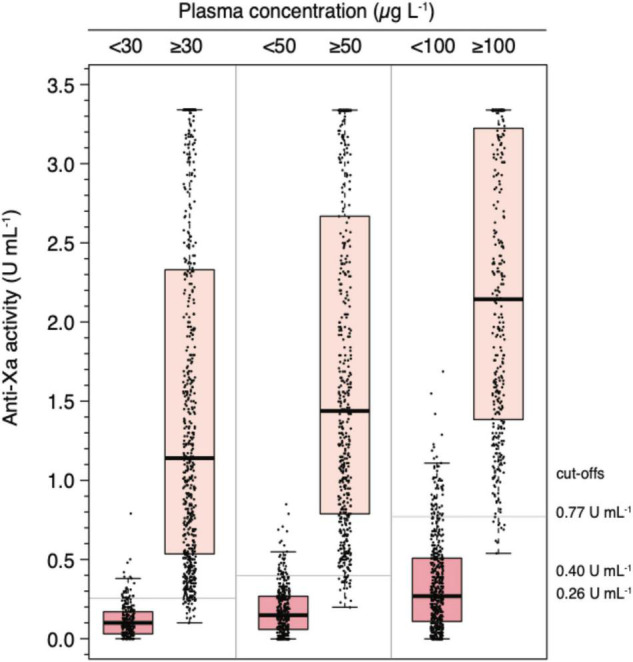
Distribution of universal, heparin-calibrated anti-Xa measurements for the measurement of rivaroxaban, apixaban, and edoxaban drug concentrations in clinical practice (*n* = 845). Box plot illustrating the distribution of measurement (median, interquartile range, minimum, maximum) at clinically relevant cut-off levels (above and below 30, 50, and 100 μg L^–1^).

The association between anti-Xa activity and drug concentrations as measured by LC-MS/MS is shown in Figure 2, and results of correlation analyses are provided in Table 2. The overall Spearman’s correlation coefficient (r_*s*_) was 0.97 (95% confidence interval, CI, 0.97 to 0.98). Regarding the individual drugs, r_*s*_ was 0.97 (95% CI 0.97–0.98) for rivaroxaban, 0.96 (0.95–0.97) for apixaban, and 0.96 (0.94–0.99) for edoxaban. The overall slope of the regression equation was 0.008 (95% CI 0.007–0.009), 0.008 (0.007–0.009) for rivaroxaban, 0.009 (0.008–0.010) for apixaban, and 0.006 (0.005–0.008) for edoxaban. The overall Y-intercept was 0.21 (95% CI 0.13–0.28), 0.29 (0.16–0.40) for rivaroxaban, 0.12 (0.016–0.022) for apixaban, and 0.10 (0.007–0.16) for edoxaban. The association between anti-Xa activity and drug concentrations over the range of measurements is shown in a modified Bland-Altman plot, see Supplementary Figure 1. The overall bias was 0.01, with a lower limit of agreement of −0.01, and an upper limit of agreement of 0.03. The distribution of anti-Xa measurements in patients with and without clinically relevant drug levels (30, 50, and 100 μgL^–1^) is shown in Figure 3.

**TABLE 2:** Accuracy of universal, heparin-calibrated anti-Xa assay with regard to drug concentration in 845 patients taking rivaroxaban, apixaban, or edoxaban in clinical practice.

	Overall	Rivaroxaban	Apixaban	Edoxaban
Spearman’s correlation coefficient (95% CI)	0.97(0.97,0.98)	0.97(0.97,0.98)	0.96(0.96,0.97)	0.96(0.94,0.99)
Deming regression Slope (95% CI)	0.008(0.006,0.009)	0.008(0.007,0.009)	0.008(0.007,0.009)	0.006(0.005,0.007)
Y-intercept (95% CI)	0.29(0.15,0.42)	0.29(0.16,0.40)	0.14(0.05,0.23)	0.10(0.015,0.18)

*Ultra-performance liquid chromatography-tandem mass spectrometry (LC-MS/MS) was used to determine drug levels. The Spearman’s correlation coefficient is given (r_s_), and the coefficients of the Deming regression.*

Sensitivity analyses considering samples below 3.34 U ml^–1^ only yielded the following results similar to the values mentioned above: r_*s*_ overall 0.97 (95%CI 0.96, 0.97), r_*s*_ rivaroxaban 0.97 (0.96, 0.97), r_*s*_ apixaban 0.96 (0.95, 0.97), and r_*s*_ edoxaban 0.96 (0.93, 0.98). The Deming regression slope was 0.01 (overall; 95% CI 0.01, 0.01), 0.01 (rivaroxaban; 0.01, 0.01), 0.01 (apixaban; 0.01, 0.01), and 0.01 (edoxaban; 0.01, 0.01). The Y-intercept was −0.01 (overall; −0.05, 0.03), −0.03 (rivaroxaban; −0.08, 0.01), −0.01 (apixaban; −0.06, 0.04), and 0.04 (edoxaban; −0.05, 0.13).

### Diagnostic Accuracy Regarding Clinically Significant Drug Levels

**FIGURE 4:**
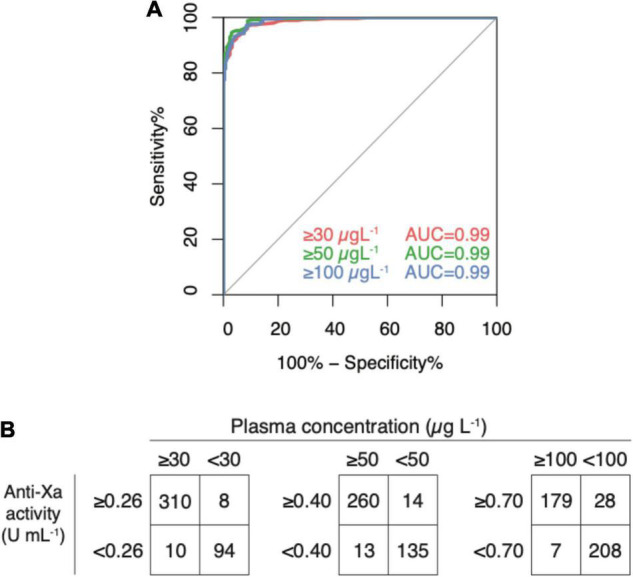
Diagnostic accuracy of a universal, heparin-calibrated anti-Xa assay for the measurement of rivaroxaban, apixaban, and edoxaban drug concentrations in clinical practice (derivation dataset; *n* = 422). **(A)** Receiver-operating characteristics curve (ROC) showing the diagnostic accuracy. The area under the ROC curve was 0.99 for all cut-offs. **(B)** Confusion matrices at each cut-off. Drug levels above 30 μg L^–1^ were detected with a sensitivity of 96.9% (95% CI 94.3, 98.5; cut-off value 0.26 U mL^–1^), drug levels above 50 μg L^–1^ with a sensitivity of 95.2% (95% CI 92.0, 97.4; cut-off value 0.40), and drug levels above 100 μg L^–1^ with a sensitivity of 96.2% (95% CI 92.4, 98.5; cut-off value 0.77). Specificities were 92.2% (85.1, 96.6), 90.6% (84.7, 94.8), 88.1% (83.3, 92.0), respectively.

In the derivation dataset (*n* = 422), the area under the ROC curve was 0.99 for all clinically relevant drug concentrations (Figure 4, panel A); 95% CI were 0.977 to 0.997 in case of 30 μg L^–1^, 0.981–0.995 in 50 μg L^–1^, and 0.978–0.994 in 100 μg L^–1^. Drug levels above 30 μg L^–1^ were detected with a sensitivity of 96.9% (95% CI 94.3–98.5; cut-off value 0.26 U mL^–1^), drug levels above 50 μg L^–1^ with a sensitivity of 95.2% (95% CI 92.0–97.4; cut-off value 0.40), and drug levels above 100 μg L^–1^ with a sensitivity of 96.2% (95% CI 92.4–98.5; cut-off value 0.77). Specificities were 92.2% (85.1–96.6), 90.6% (84.7–94.8), 88.1% (83.3–92.0), respectively. Confusion matrices are given in Figure 4, panel B. In the sensitivity analyses, these measures did not differ significantly.

### Internal Validation

**FIGURE 5:**
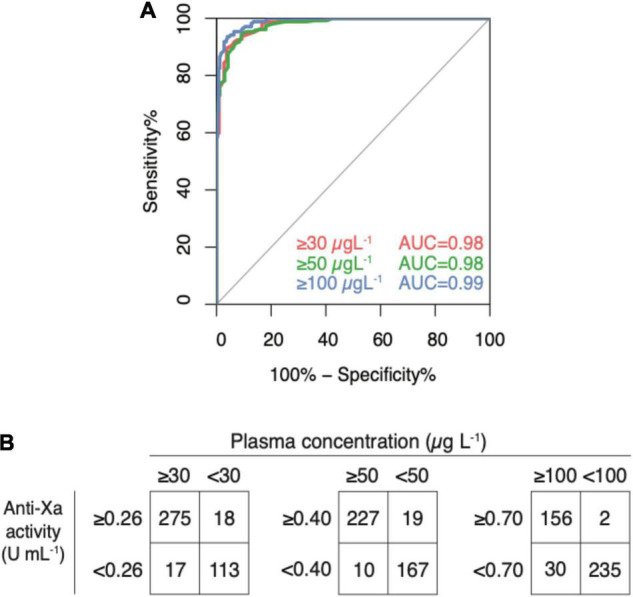
Internal validation of a universal, heparin-calibrated anti-Xa assay for the measurement of rivaroxaban, apixaban, and edoxaban drug concentrations in clinical practice (verification dataset; *n* = 423). **(A)** Receiver-operating characteristics curve (ROC) showing the diagnostic accuracy. The area under the ROC curve was 0.98 for both the cut-offs 30 and 50 μg L^–1^ and 0.99 for 100 μg L^–1^. **(B)** Confusion matrices at each cut-off. Drug levels above 30 μg L^–1^ were detected with a sensitivity of 94.2% (95% CI 90.8, 96.6; cut-off value 0.26 U mL^–1^), drug levels above 50 μg L^–1^ with a sensitivity of 95.8% (95% CI 92.4, 98.0; cut-off value 0.40), and drug levels above 100 μg L^–1^ with a sensitivity of 98.7% (95% CI 95.5, 99.9; cut-off value 0.77). Specificities were 86.3% (79.2, 91.7), 89.8% (84.5, 93.7), 88.7% (84.2, 92.2), respectively.

In the verification dataset (*n* = 423), the area under the ROC curve was 0.98 for both the cut-offs 30 (0.971–0.990) and 50 (0.976–0.993) μg L^–1^ and 0.99 (0.988–0.998) for 100 μg L^1^ (Figure 5, panel A). Cut-off levels obtained in the derivation dataset were used to calculate sensitivities and specificities in the verification dataset (*n* = 423) as an internal validation (Figure 5, panel B). Drug levels above 30 μg L^–1^ were detected with a sensitivity of 94.2% (95% CI 90.8–96.6; cut-off value 0.26 U mL^–1^), drug levels above 50 μg L^–1^ with a sensitivity of 95.8% (95% CI 92.4–98.0; cut-off value 0.40), and drug levels above 100 μg L^–1^ with a sensitivity of 98.7% (95% CI 95.5–99.9; cut-off value 0.77). Specificities were 86.3% (79.2–91.7), 89.8% (84.5–93.7), 88.7% (84.2–92.2), respectively. In the sensitivity analyses, these measures did not differ significantly.

## Discussion

We conducted a large multicenter cross-sectional study including 845 patients treated with rivaroxaban, apixaban, or edoxaban in clinical practice. Even though minor differences between drugs and some variability in the higher concentrations exist, the association between heparin-calibrated anti-Xa measurements and LC-MS/MS results was strong for all drugs. As an internal validation, clinically relevant drug levels were predicted correctly in the verification dataset using cut-off levels derived in the derivation dataset.

Even though this is the first study analyzing a UFH-calibrated anti-Xa assay in a large cohort, our results are essentially in-line with previous publications. Recently, we analyzed the accuracy of a LMWH-calibrated anti-Xa assay in the same cohort (*n* = 867) (21). Similarly, the accuracy was high and clinically relevant drug levels were predicted correctly. Studt et al. (17) studied the accuracy and consistency of anti-Xa assays for rivaroxaban plasma concentration in 20 healthy individuals and found a high agreement with drug concentrations measured by LC-MS/MS. Another cross-sectional study (25) showed a strong correlation between heparin anti-factor Xa activity and LC-MS/MS in 30 patients treated with rivaroxaban. In another retrospective study, a high degree of correlation between a heparin-calibrated anti-Xa assay and LC-MS/MS was observed in 24 patients taking rivaroxaban or apixaban (26). Similar results were also observed in studies assessing the correlation of anti-Xa measurements with LC-MS/MS in spiked samples (27–30). A high correlation between UFH-calibrated anti-Xa measurements and rivaroxaban-/apixaban-calibrated anti-Xa results was found in a study assessing 241 left-over samples (20). Besides, van Pelt and colleagues proposed a universal anti-Xa assay reporting the inhibitory effect rather than drug concentrations (18).

This study is associated with certain strengths and limitations. The sample size is much larger than previous studies (*n* = 845), which increases statistical power and precision. Thus, we were able to split the dataset into a derivation and verification dataset, facilitating internal validation of the results. Another strength is that our study was designed as a multicenter study (nine laboratories) conducted in clinical practice, thus constituting a representative population. Therefore, the results can be translated straightforwardly to clinical practice. Also, LC-MS/MS was used as a reference standard which is considered the most accurate technique to measure DOAC plasma levels (21, 31). As a limitation, only a single heparin-calibrated assay was studied and other heparin-calibrated anti-Xa assays may perform differently. Several previous studies using various study designs found considerable differences among reagents (19, 32, 33). Additionally, the number of edoxaban-treated patients was lower compared to rivaroxaban and apixaban-treated patients. However, we believe that this is compensated by the large sample size.

The question now arises as to how this test can be applied in daily practice. The cut-off values mentioned in Table 3 can be given so that clinical decisions can be prompted directly (Table 3). Alternatively, the regression equations can be used to calculate the plasma concentrations of each compound (Table 3). However, the second approach must be used with caution. Minor differences between drugs exist and a certain degree of variability can be observed in higher concentrations. Therefore, drug levels in high concentrations (≥150 μgL^–1^) can only be measured with limited accuracy and precision (34). Yet, several arguments can be raised in favor of the heparin-calibrated assay: (1) this variation is also present in drug-specific anti-Xa assays (17), (2) typical requirements in terms of correlation coefficients and ROC AUC are fulfilled, and (3) all clinically relevant cut-off thresholds are met with high accuracy. Current guidelines do not distinguish between normal-high and very-high drug levels concerning patient management.

**TABLE 3:** Implementation of a single, heparin-calibrated anti-Xa assay for the measurement of rivaroxaban, apixaban, and edoxaban drug concentrations.

	Cut-off value	Regression equation
**Clinical threshold**		
30 μg/L DOAC	0.26 U/mL	
50 μg/L DOAC	0.40 U/mL	
100 μg/L DOAC	0.77 U/mL	
**Drug concentrations**		
Rivaroxaban		129 × [U/mL] – 37
Apixaban		124 × [U/mL] – 17
Edoxaban		166 × [U/mL] – 16

*The cut-off values could be given, which would allow direct clinical decisions to be made. Alternatively, the regression equations can be used to calculate the plasma concentrations of each compound.*

Our results confirm that a single, heparin-calibrated anti-Xa assay accurately measures rivaroxaban, apixaban, and edoxaban drug concentrations and correctly predicts clinically relevant drug levels. Since heparin-calibrated anti-Xa assays are already available in many laboratories, determination of DOAC drug levels can be provided easily. This may foster the widespread implementation of anti-Xa assays to measure DOAC, thus improving care in patients taking these drugs. Future studies should confirm these results in other settings and using other heparin-calibrated assays.

### Conclusion

We report results of a large prospective study including patients treated with rivaroxaban, apixaban, or edoxaban in clinical practice. The association of heparin-calibrated anti-Xa measurements with DOAC drug concentrations was strong, and clinically relevant drug levels were predicted correctly. Our results represent a strong argument in favor of the potential application of a universal, heparin-calibrated anti-Xa assay to measure DOAC in clinical practice.

## Data Availability Statement

The raw data supporting the conclusions of this article will be made available by the authors, without undue reservation.

## Ethics Statement

The studies involving human participants were reviewed and approved by the Kantonale Ethikkommission Bern. The patients/participants provided their written informed consent to participate in this study.

## Author Contributions

TM analyzed the data, interpreted the results, and wrote the manuscript. J-DS, AM, LAl, PF, WW, AS, LG, BG, CB, LAs, UA, and TS collected data, and contributed to study design, protocol, and preparation of the manuscript. CB and UA contributed essential tools and reagents. MN designed the study, wrote the protocol, collected data, analyzed, and interpreted the data, and wrote the manuscript. All authors contributed to the article and approved the submitted version.

## Conflict of Interest

MN reports research grants from Bayer Healthcare, outside of the submitted work, lecture honoraria from Bayer Healthcare, and Daiichi Sankyo. LAl reports research grants from Bayer, CSL-Behring, Novartis, Novo Nordisk, Roche, Sobi, and Takeda. WW reports research grants from Bayer Healthcare, BMS-Pfizer, Daiichi Sankyo and Sanofi, and honoraria for participating in scientific advisory boards from Bayer, Pfizer, and from Alexion Pharma GmbH, all outside the submitted work. J-DS reports lecture fees and advisory honoraria from Bayer Healthcare, Pfizer, Takeda, Siemens, and Sanofi. TS holds an endowed professorship supported by the Touring Club Switzerland. BG reports non-financial support and funding for accredited continuing medical education program from Axonlab, and from Thermo Fisher Scientific, during the conduct of the study; personal fees and funding for accredited continuing medical education program from Alnylam, grants, personal fees and funding for accredited continuing medical education program from Pfizer, funding for accredited continuing medical education program from Bayer, Bristol Myers Squibb, Daiichi Sankyo, Takeda, Octapharma, SOBI, Janssen, Novo Nordisk, Mitsubishi Tanabe Pharma, outside the submitted work. The remaining authors declare that the research was conducted in the absence of any commercial or financial relationships that could be construed as a potential conflict of interest.

## Publisher’s Note

All claims expressed in this article are solely those of the authors and do not necessarily represent those of their affiliated organizations, or those of the publisher, the editors and the reviewers. Any product that may be evaluated in this article, or claim that may be made by its manufacturer, is not guaranteed or endorsed by the publisher.

## Supplementary Material

Supplementary Figure 1 A modified Bland–Altman plot using ratios to determine a possible systematic bias over the range of measurements. The bias and the upper and lower limit of agreement is shown.
