# Supplementary figures and images for: Accuracy of a Single, Heparin-Calibrated Anti-Xa Assay for the Measurement of Rivaroxaban, Apixaban, and Edoxaban Drug Concentrations: A Prospective Cross-Sectional Study
Source: Front Cardiovasc Med. 2022 Mar 17;9:817826. doi: 10.3389/fcvm.2022.817826 (PMC8969025; doi:10.3389/fcvm.2022.817826)

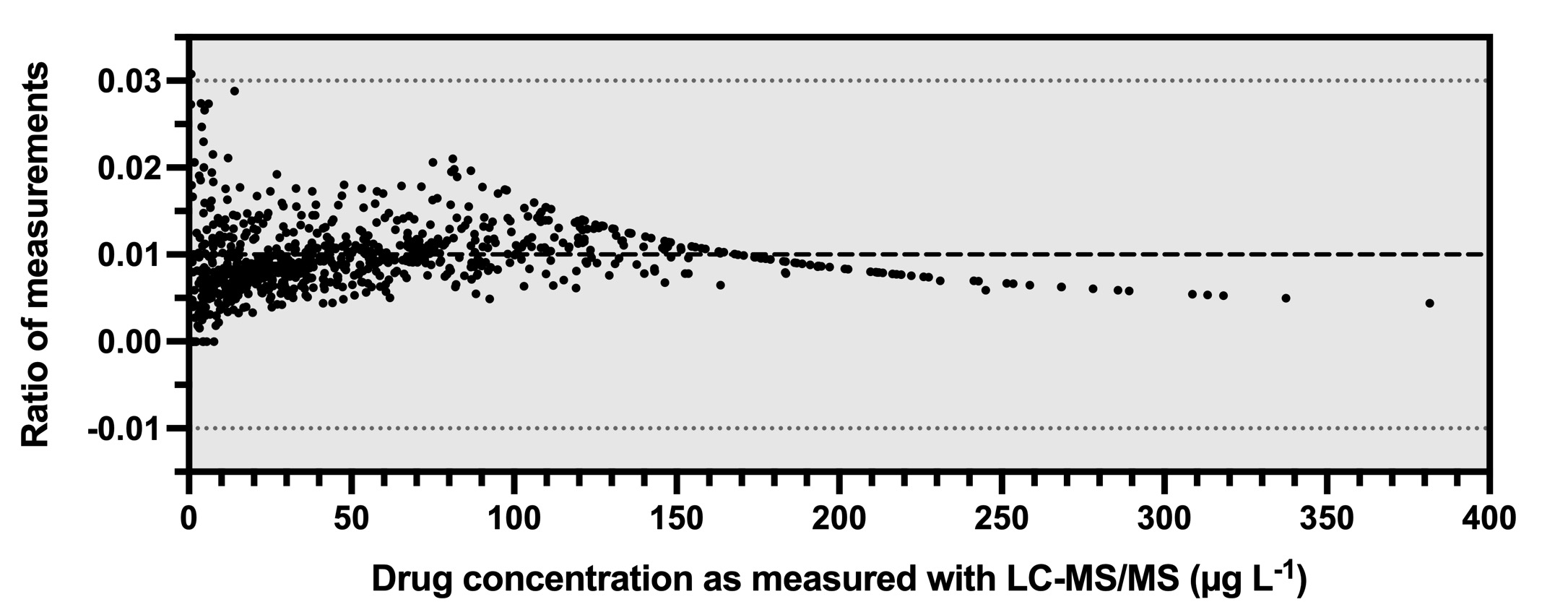

Supplement: Supplementary Figure 1 — A modified Bland–Altman plot using ratios to determine a possible systematic bias over the range of measurements. The bias and the upper and lower limit of agreement is shown. [file Image_1.jpg]
